# Social Media Use in Research: Engaging Communities in Cohort Studies to Support Recruitment and Retention

**DOI:** 10.2196/resprot.4260

**Published:** 2015-07-22

**Authors:** Eva Farina-Henry, Leo B Waterston, Laura L Blaisdell

**Affiliations:** ^1^ Center for Outcomes Research and Evaluation Maine Medical Center Research Institute Portland, ME United States

**Keywords:** social media, longitudinal studies, pilot project, community outreach

## Abstract

**Background:**

This paper presents the first formal evaluation of social media (SM) use in the National Children’s Study (NCS). The NCS is a prospective, longitudinal study of the effects of environment and genetics on children’s health, growth and development. The Study employed a multifaceted community outreach campaign in combination with a SM campaign to educate participants and their communities about the Study. SM essentially erases geographic differences between people due to its omnipresence, which was an important consideration in this multi-site national study. Using SM in the research setting requires an understanding of potential threats to confidentiality and privacy and the role that posted content plays as an extension of the informed consent process.

**Objective:**

This pilot demonstrates the feasibility of creating linkages and databases to measure and compare SM with new content and engagement metrics.

**Methods:**

Metrics presented include basic use metrics for Facebook as well as newly created metrics to assist with Facebook content and engagement analyses.

**Results:**

Increasing Likes per month demonstrates that online communities can be quickly generated. Content and Engagement analyses describe what content of posts NCS Study Centers were using, what content they were posting about, and what the online NCS communities found most engaging.

**Conclusions:**

These metrics highlight opportunities to optimize time and effort while determining the content of future posts. Further research about content analysis, optimal metrics to describe engagement in research, the role of localized content and stakeholders, and social media use in participant recruitment is warranted.

## Introduction

In 2000, Congress authorized the National Institutes of Health to conduct the National Children’s Study (NCS); a prospective, longitudinal study of US children and their parents, designed to examine the effects of environment and genetics on children’s health, growth and development. The NCS Vanguard (pilot) Study began in 2009, evaluating methods for the larger Main Study, including community engagement techniques to increase recruitment and retention of participants [1-3]. Historically, recruitment of participants for population-based, longitudinal studies has presented many challenges [4-7]. Young adults, including women of childbearing age (the target demographic for the NCS), represent a particularly challenging cohort for recruitment and retention due to their increased mobility [8-11]. Social media (Facebook, Twitter, blogs, YouTube) is increasingly a first information source for this demographic group, and has been shown to be an effective tool for participant retention in longitudinal research [12-15]. Social media circumvents geographic differences between people and provides a cost effective, convenient method of study recruitment and retention [11,13,15]. Social media also provides an interactive platform and encourages the free sharing of information.

However, using social media in biomedical research raises important considerations. Utilizing social media in the highly regulated clinical research environment requires a nuanced understanding of potential threats to confidentiality and privacy and the role that posted content plays as an extension of the informed consent process (eg one must avoid overpromising benefits or underestimation of risks) [16,17]. Additionally, institutional review boards require that researchers make every effort to minimize human subject risks when engaging participants via social media [16].The regulatory review process can slow posting frequency to a pace that threatens timeliness, relevance and response which are strengths of social media use.

This paper presents the first formal evaluation of social media use in the NCS. While few metrics exist to guide best practices for the use and evaluation of social media in the NCS and for biomedical research generally, we present here a pilot project testing the feasibility of collecting and analyzing social media metrics from multiple sites with the primary goal of determining best practices for social media use in research engagement.

## Methods

### Participants

Study Centers (SCs) utilizing Facebook as part of their community outreach and engagement efforts were invited to participate. Four SCs (Queens County, New York; Waukesha County, Wisconsin; San Diego County, California; and Cumberland County, Maine) elected to participate thus providing geographic diversity.

Participating SCs collected 4 months of data between December 2011 and March 2012 from various social media accounts and websites (see Table 1). Only Facebook data is presented in this paper because it was the only modality consistently used by all of the participating locations. SCs met via 4 monthly conference calls to report and discuss social media use. All SCs downloaded their Facebook data and provided it to the MSC for analysis.

### Metrics

We used several metrics in our Facebook analysis. We started with basic Facebook use metrics including lifetime number of likes, average likes per month (number of lifetime likes divided by the number of months on Facebook) and number of posts. At the time of this analysis, EdgeRank score (see Textbox 1) was collected as an effectiveness measure because it is an algorithm developed by Facebook to govern what is displayed on the News Feed [18]. The EdgeRank formula includes the variables of affinity, time, and weight [19]. An EdgeRank score of 20 or higher is generally considered by social media experts as positive for potential exposure in the News Feed [20].

A multitude of metrics exist in Facebook to describe engagement but at the time of this analysis there was no standard metric for engagement with content. We conducted a literature search of existing Facebook metrics for assessing user engagement using Facebook Insights data [21-24]. Although no single common metric exists, a combined Insights data set is commonly used, albeit with distinctly different approaches. Facebook collects deidentified data for pages, as well as for individual posts (see Textbox 1)*.* Page level data describes how users engage with the Facebook page, offering cumulative metrics for fans, reach, and engagement. Post level data is collected for each individual post, yielding a more focused assessment of what prompts fans (users who have liked the Facebook page) to interact with individual posts.

In page level analyses, the rate of Page Consumption (number of page clicks or video views) over Total Daily Page Reach (number of people who saw your page content) is commonly used as an engagement metric, however Page Consumption does not include post likes, shares, or comments. In Post level analyses, current engagement metrics measure the rate of comments and likes on a post over the number of impressions (number of times a post is displayed, regardless of whether it is seen or not in the News Feed).

The Facebook pages used in this study had a relatively low number of page likes. SC Facebook pages count likes in the mid to low hundreds compared to commercial products that count fans in the millions (Nike has over 22 million fans). The engagement metrics for Page and Post level Insights described above did not accurately describe the activity we saw on the pages. To better understand how our fans engaged with our posts, we determined that it was essential to include all activities of engagement when analyzing our data. This included any interaction between a fan and a post—any click on a post, like, comment, or share. We chose to use Post Total Reach in our engagement analysis as well. Post Total Reach is the total number of unique people who see the post in their News Feed, as defined and measured by Facebook.

**Table 1:** Study center characteristics and social media use by location.

Study center location	Social media modalities used	Months on Facebook	Rural/Urban	Population (US Census 2010)
Queens County, New York	Facebook, Twitter, YouTube, Blog	12	Urban	2,230,722
Waukesha County, Wisconsin	Facebook	18	Rural	389,891
Cumberland County, Maine	Facebook, Twitter, Blog	14	Rural	281,674
San Diego County, California	Facebook, Twitter	17	Urban	3,095,313

**Key Facebook analytic terms.**

EdgeRank - As of 2011, the Facebook algorithm that determined what is displayed on a user’s News Feed [19]

Page level data - Data that provides an overview of the overall Page performance and metrics related to change in audience [13]

Post level data - Data about a particular post [13]

Reach - The number of persons who have seen content associated with your Page [13]

Consumption - The number of clicks or video views [13]

Impressions - The number of times a post is displayed, regardless of whether it is seen in a News Feed or not [13]

### Analysis

Content analysis examines the topics that SCs posted about during the study period. Since Facebook does not collect metrics about the content of posts, we developed a unique analysis, conducted by collecting SC posts and assigning each post to one of nine content categories, classifying the overall intent of each post (see Textbox 2). Inter-rater reliability testing of post-content classification was performed with four raters assigning posts to categories and produced a kappa ranging from 0.67-0.75 indicating adequate inter-rater agreement.

**Figure 1:**
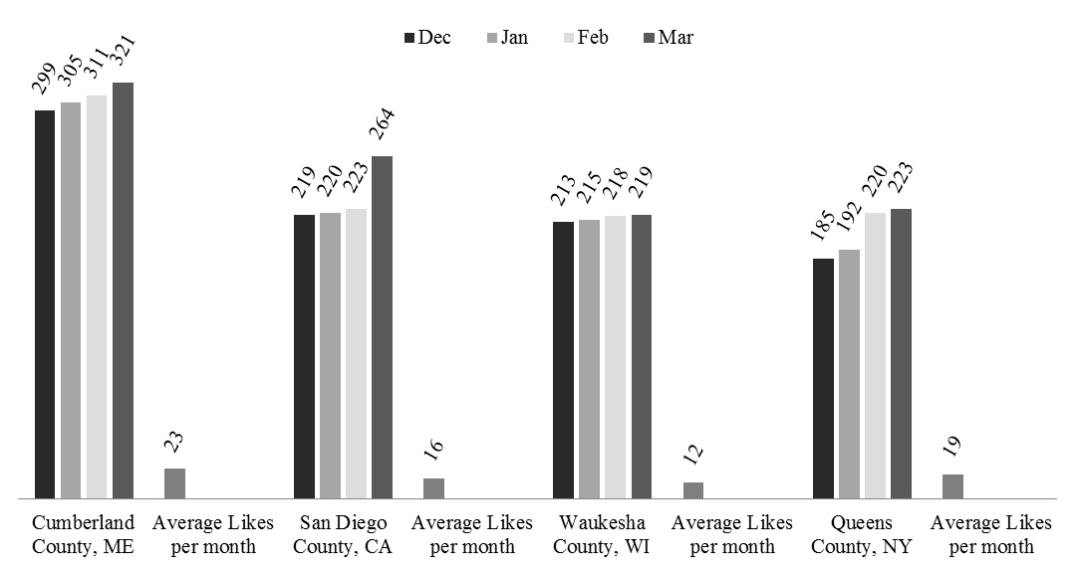
Facebook cumulative Likes over a 4 month period and average number of likes per month for the 4 participating SCs.

By assigning each post to one category, the prevalence, or frequency of a SC posting about that topic was calculated. Prevalence of each post category was represented as a percentage of the total posts (see Figure 1). Using the lifetime total reach and lifetime engaged user metrics from each post, an overall total reach and engagement was summed for each content category. The engagement score was then calculated by dividing the total engagement for a category by its total reach. This method of analysis was applied to each study center.

This project was declared exempt from review by the Maine Medical Center Institutional Review Board. All social media activity strictly followed NCS social media policies [17,25].

**Categories used to analyze the topics of posts.**

National Children’s Study (including updates, newsletters, publications)

Holidays

Activity (focusing specifically on local or regional events)

Kids (content directly related to or for children)

Health and fitness (focusing on physical fitness and active living)

Health education

Nutrition

Education (including parenting)

Awareness (health observances and monthly causes)

## Results

We present here both basic use metrics for Facebook, as well as the Facebook content and engagement analysis described above.

### Basic Use Metrics for Facebook

**Figure 2:**
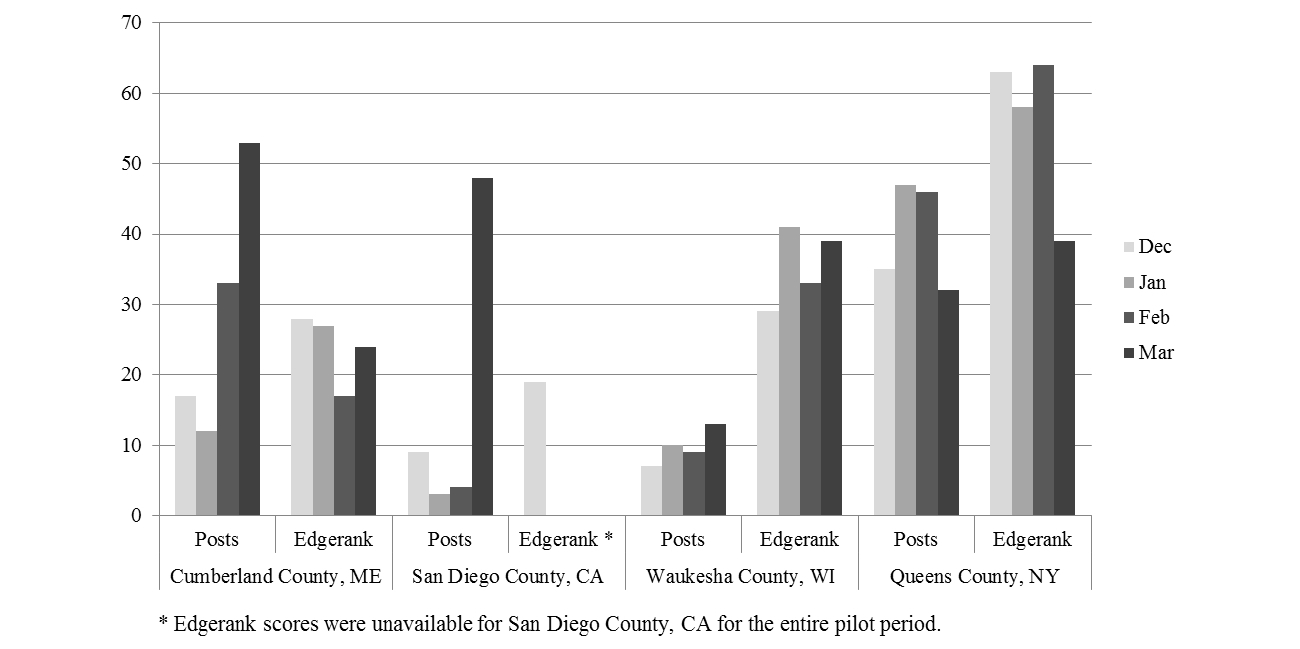
Number of posts and EdgeRank scores for the study period by month and SC.

The 4 sites experienced an average of 229 (range 185-299) Facebook Likes in December 2011; by the completion of the pilot in March 2012, this average jumped to 257 (range 219-321), an average increase of 28 likes (range 6-45) over the pilot period. Likes is a cumulative metric over the period of Facebook use, therefore we calculated “Likes per Month”. The SC Facebook pages averaged 16 (range 12-18) months of publishing and the Likes per Month ranged from 12 to 23 (see Figure 2). SCs averaged 17 posts per month (range 7-35) and generally increased to 37 posts per month (range 13-53) over the study period.

**Figure 3:**
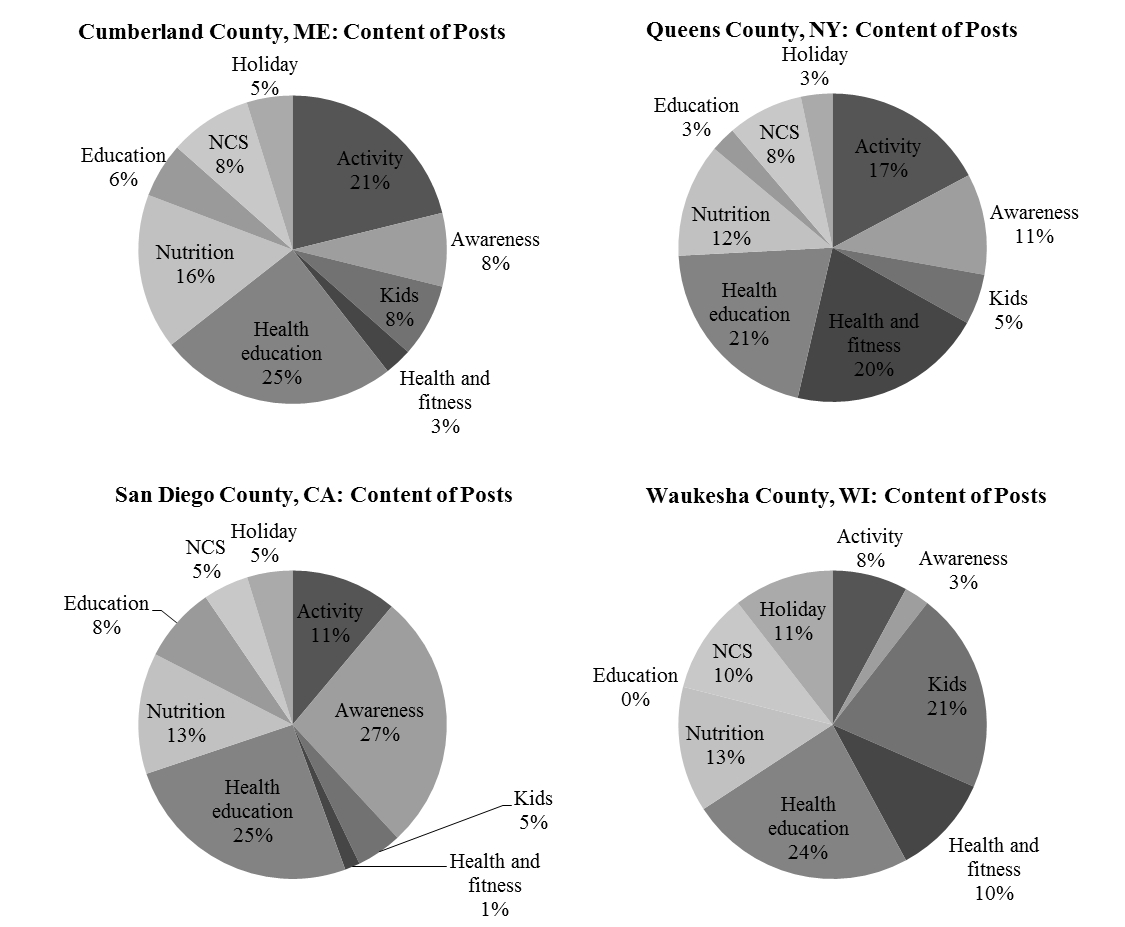
Content posted by each Study Center over the pilot period.

EdgeRank scores for the strength of post placement in the News Feed were variable over the sites, as shown in Figure 3, and the monthly average by site ranged between 34 and 42 (see Figure 3). We expect a positive correlation between EdgeRank scores and number of posts [19,20]. Waukesha County, WI and Queens County, NY demonstrates this relationship, however, Cumberland County, ME does not. As the number of posts increases in Cumberland County, ME, the EdgeRank score decreases.

### Facebook Content Analysis and Engagement Analysis

For each SC we analyzed the post content throughout the pilot period (Figure 1). Analysis of post content showed that SCs posted most commonly about health education (24.3%, (range 21%-27%)), activity (14.3%, (range 8%-21%)), and nutrition (13.8%, (range 13%-16%)). Content of showed some variability between SCs. For example, San Diego County, CA posted mostly about health awareness issues (27%), while Waukesha County, WI posted most frequently about children’s topics (21%).

Table 2 shows the engagement analysis for each Study Center by content category. We calculated the overall engagement per view as well as the overall views per post for each Study Center. There was some variation in the views per post (ranging from 66.095-84.875) and engagement per view (range 0.053-0.039). Posts about the NCS have high engagement per view across all Study Centers. Posts about holidays had the highest views in Waukesha County, WI, while posts about nutrition had the most views per post in Cumberland County, ME. Additionally, some Study Centers had high engagement on activity and nutrition-related content. Content analysis demonstrated overall low engagement scores with health education topics, but regional variation was seen with other topics. Waukesha experienced high engagement on posts related to activity or awareness, but the other sites had significantly lower engagement in these categories. Interestingly, three sites (Cumberland County, ME; Queens County, NY; and San Diego County, CA) experienced higher engagement with child related content, but Waukesha County, WI experienced little engagement relative to the number of posts about this content.

Interestingly, in Cumberland County, ME, we notice an inverse relationship between views per post and engagement per post. When looking at the overall engagement in Cumberland County, ME, (Table 2) many people saw each post as reported by the high views per post, yet few people engaged with post as indicated by the lowest rate of engagement per view. This pattern supports an acknowledged relationship in Facebook where as a Page reaches more people, a lower proportion of that audience will engage with content. By comparing the engagement of each content category to the overall engagement, we can derive which topics are of most interest to each Study Center and tailor social media content to local community interests.

**Table 2:** Content engagement variance as a function of Study Center.

		Overall content engagement variance	By Content Category								
			Activity	Awareness	Kids	Health and fitness	Health education	Nutrition	Education	NCS	Holiday
Cumberland County, ME	Engagement per view	0.039	0.052	0.019	0.045	0.034	0.031	0.040	0.029	0.052	0.034
	Views per post	84.875	80.591	86.125	81.375	88.667	88.308	90.176	80.833	77.444	87.400
Queens County, NY	Engagement per view	0.040	0.044	0.030	0.032	0.031	0.035	0.034	0.042	0.093	0.046
	Views per post	82.079	87.654	79.471	82.125	82.071	78.563	83.842	76.750	83.917	77.600
Waukesha County, WI	Engagement per view	0.040	0.032	0.039	0.038	0.030	0.035	0.045	0.000	0.062	0.034
	Views per post	75.778	73.000	76.000	78.000	66.500	76.900	76.200	0.000	68.500	87.000
San Diego County, CA	Engagement per view	0.053	0.048	0.055	0.080	0.019	0.040	0.064	0.044	0.103	0.030
	Views per post	66.095	54.000	71.176	67.000	54.000	62.563	74.750	68.600	71.000	55.333

## Discussion

### Principal Findings

Social media use is new in research recruitment and retention and holds significant potential, however there are currently no well defined best practices and metrics for evaluation.

We describe here a pilot project testing the feasibility of data collection through Facebook, as well as the creation and analysis of social media metrics from multiple SCs with the primary goal of determining best practices for future use. Primarily, this pilot demonstrated the feasibility of creating linkages and databases to collect and measure social media metrics from multiple SCs for comparison of content and engagement. Shared posting schedules and best practices combined with coordinated engagement (eg liking each other’s posts to increase reach) contributed to a measurable increase in engagement during the study period.

Facebook was the only social media modality evaluated in this pilot study. Assessment of engagement across SCs and modalities was complicated by a lack of commonly used and/or accepted metrics. We describe here basic Facebook metrics, along with the nascent creation of content and engagement analysis metrics tailored for NCS evaluation. Likes are commonly used as a measure of engagement; however, likes are garnered and not often lost. Therefore likes are a cumulative, static metric and not a moving metric like engagement. Number of likes does not indicate engagement, but does represent a “fan base” and potential for engagement. The difference in likes per month demonstrates that these online communities can be quickly generated (see Figure 2). For example, Cumberland County, Maine was able to yield a similar number of likes as the two NCS locations that were the most established on Facebook in a shorter amount of time, although other factors (eg authorization for mass communications campaigns and the use of social media) must be considered in interpretation. Because of the limitations of using likes in measuring engagement, we describe here the evaluation of new engagement metrics.

EdgeRank is commonly used to measure overall success and impact on Facebook, however it does not allow analysis of the content of posts, nor does it inform on how to serve interesting and engaging content to individual communities. The algorithm that EdgeRank uses includes variables of affinity, time, and weight [18-20]. Cumberland County, Maine’s EdgeRank scores continued to be low despite posting significantly more frequently in the last study month, which, as a variable in EdgeRank’s algorithm, should have yield an increased score (see Figure 3). This example highlights the challenges of using this algorithm to see and respond to our community’s variable interests in content.

Content and engagement analysis yielded new and improved metrics for the use of social media in the NCS. These analyses allowed us to describe what content of posts SCs were using, what content they were posting about, and what the online NCS communities found most engaging (see Figure 1). Engagement metrics shed light on where to focus time and effort in determining the content of posts (see Table 2). The purpose of this study was to demonstrate the ability to measure differences in engagement, yet an important realization is that not all centers should necessarily post the same content—rather the content posted should reflect their community and study activity. Questions remain; What messages were different between centers when it came to the topics of children? What role does localized content play in engagement on Facebook? Future research could further explore the role of content on engagement, as well as the optimal metrics to describe engagement in the research setting.

### Limitations and Challenges

This pilot project had several limitations and challenges. This pilot study was not a representative sample of all SCs. The study proposed new metrics that lack baseline data to which to compare our engagement scores, making it difficult to interpret the data relative to other Facebook sites. We minimized subjectivity through inter-rater analysis, however, subjectivity in content categories and the sorting of posts must be acknowledged. The use of social media in the research setting is necessarily restricted by regulations for human subject protections. As such, we were limited in the content we could post and were unable to directly solicit engagement from our target market— NCS participants— and instead targeted the general community corresponding to each Facebook site. While ethical review of using social media to support the conduct of research is evaluated by federal restrictions through institutional review, many social media users neither read nor understand complex social media terms of service agreements and may not see themselves as potential research subjects. This latter point will continue to be a source of challenge for this field of study.

### Conclusions

This pilot developed the infrastructure to analyze social media use in 4 SCs in the NCS, as well as demonstrating some preliminary best practices. Our analysis supports the use of social media, specifically Facebook, to increase awareness of the Study. Facebook’s strengths include the ability to engage in a two way dialogue and the open sharing of information, but understanding how best to utilize this forum’s user base and strengths for the changing goals and regulations of the use of social media in research. Further research examining content analysis, the role of localized of content and stakeholders, and social media use in participant recruitment is warranted.

## Abbreviations

NCS: National Children’s Study
MSC: Maine Study Center
SC: study center
SM: social media
